# Evaluation of the Effectiveness of a Structured Educational Program on Improving the Knowledge of COVID-19 Among the Vaccine-Reluctant Population in a Block of Jharkhand, India

**DOI:** 10.7759/cureus.38628

**Published:** 2023-05-06

**Authors:** Muskan Kumari, Himel Mondal, Gujaram Marndi, Ayesha Juhi

**Affiliations:** 1 Physiology, All India Institute of Medical Sciences, Deoghar, Deoghar, IND; 2 Pharmacology, Dharanidhar Medical College and Hospital, Keonjhar, Kendujhar, IND

**Keywords:** local language, jharkhand, hesitancy, awareness, educational program, immunization programs, vaccination, vaccines, covid-19

## Abstract

Background

Low vaccination uptake is a major public health concern and is more prevalent in rural areas. Educational interventions have been proposed as an effective strategy to increase vaccine acceptance. The objective of this study was to assess the impact of an educational program on acquiring knowledge for promoting vaccination uptake among a sample of participants.

Methodology

This study was conducted in a rural area in the state of Jharkhand, India. The study period was from July 2022 to September 2022. The area was surveyed for COVID-19 vaccination and a total of 510 people did not take any dose or took only the first dose and then skipped the second dose. An educational program was designed in the local language. The knowledge of the sample was assessed before and after a week of intervention with a surveyor-administered questionnaire. The vaccination status before and after the intervention was also recorded. We used the chi-square test, Fisher’s exact test, and binomial test for comparing the categorical variables.

Results

A total of 178 participants’ data were analyzed. The majority of the participants were in the age group of 18-25 years. The pre-intervention score regarding the knowledge of COVID-19 and vaccination was 18.93 ± 5.10 which significantly increased after the intervention to 25.06 ± 4.35 (p <0.0001). The number of individuals receiving vaccination significantly increased. Before the program, 95 participants did not take the vaccine and 83 received the first dose and did not take the second dose. After the program, 17 participants did not take the vaccine, 161 completed the first dose, and 112 completed the second dose (p <0.0001).

Conclusions

The educational program was successful in improving knowledge and awareness about vaccination, leading to an increase in the number of individuals receiving vaccination. These findings suggest the importance of educational interventions in the local language in promoting vaccination uptake and can be used to design effective public health campaigns to increase vaccine acceptance.

## Introduction

The COVID-19 pandemic has affected populations globally, and vaccination has been identified as one of the most effective measures to control its spread [1]. However, vaccine hesitancy has emerged as a significant challenge to achieving herd immunity. In particular, certain populations have expressed reluctance to receive the vaccine due to a lack of knowledge about its safety and efficacy [2]. As vaccination remains crucial in reducing severe disease, hospitalizations, and deaths and safeguarding the unvaccinated from new variants, we need to develop evidence-based campaigns that effectively address vaccine hesitancy in rural villages, including tribal populations [3].

Educational programs play a vital role in removing vaccine hesitancy by providing accurate and reliable information about the safety and efficacy of vaccines. These programs are designed to address common misconceptions and myths that contribute to vaccine hesitancy and to provide a clear understanding of the benefits of vaccination [4]. Moreover, these programs can be tailored to address the specific concerns of vaccine-hesitant populations, including those living in rural or remote areas, thereby increasing their accessibility and effectiveness [5].

In this context, this study aimed to evaluate the effectiveness of a structured educational program in improving knowledge and reducing vaccine hesitancy regarding COVID-19 vaccination among the vaccine-reluctant population residing in a block of Jharkhand, India.

## Materials and methods

Before conducting this study, ethical clearance was obtained from the Institutional Ethical Committee of the All India Institute of Medical Sciences, Deoghar, Jharkhand, India (reference number: 2022-55-EMP-02 [STS 2022-01489]). This was an interventional study conducted among a non-vaccinated or partially vaccinated adult population residing in Devipur Block, Deoghar district, Jharkhand. The study location is in the eastern part of India. The study was conducted from July 2022 to September 2022.

The sampling method used was cluster sampling, and the study subjects were selected from 17 panchayats falling under the Community Health Centre, Devipur. A cluster of 30 subjects from each panchayat was selected, resulting in a sample size of 510 individuals. The list of individuals who were not vaccinated was obtained from the Block Medical Officers, Auxiliary Nurse Midwife, and Accredited Social Health Activist workers responsible for different panchayats.

**Figure 1:**
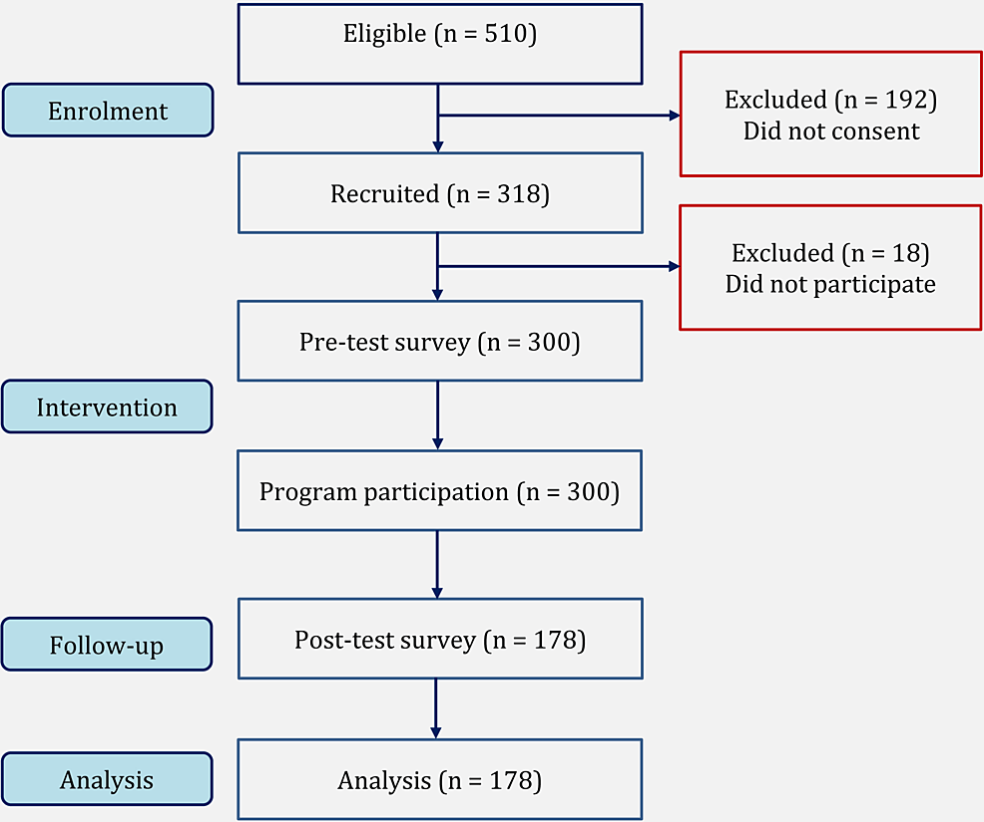
Number of participants in different stages of the study.

Inclusion criteria included adults above 18 years of age who had not received even a single dose of the COVID-19 vaccine or those who had received the first dose but had not taken the second dose beyond the scheduled gap of the second dose. Written consent was obtained from the study subjects after explaining the details of the study. The number of research participants is shown in Figure 1.

A questionnaire was prepared by six experts including three public health experts, one physiologist, one microbiologist, and one pathologist. The questionnaire was prepared following the modified Delphi method. It was pre-tested on 30 participants. The questionnaire (Appendix 1) was used as a pre-test to assess the subject’s knowledge about the COVID-19 vaccine. The survey was conducted in the local language by an expert surveyor recruited from the locality. This was followed by a structured educational program conducted in the form of a video (content overview is available in Appendix 2) and pictures in a batch of 10-15 in a common area. The video was made in the local language for better dissemination of awareness about the importance of COVID-19 vaccination. A question-and-answer session was conducted with the study participants and investigators for clearing any doubts. Finally, a post-test questionnaire was administered the day after the educational program to assess the subjects’ knowledge about the COVID-19 vaccine.

Statistical analysis

Paired t-test was performed to determine if there was a statistically significant difference between the means of pre-test and post-test scores of the answers provided by the participants. We used the chi-square test, Fisher’s exact test, or binomial test for comparing the categorical variables. A p-value of less than 0.05 was considered statistically significant. We used GraphPad Prism 7.0 for conducting statistical tests.

## Results

A total of 178 participants’ data were analyzed. The demographics of the study population are shown in Table 1. The majority of the participants were in the age group of 18-25 years.

**Table 1:** Demographic details of the research participants (n = 178). *: Statistically significant p-value of the chi-square test or Fisher’s exact test; †: Statistically significant p-value of the binomial test.

Variable	Category	Values (%)	P-value
Age (years)	18–25	67 (37.64)	<0.0001*
	26–35	42 (23.6)	
	36–45	27 (15.17)	
	46–55	22 (12.36)	
	56–65	13 (7.3)	
	>65	7 (3.93)	
Sex	Male	103 (57.87)	0.043†
	Female	75 (42.13)	
Marital status	Married	116 (65.17)	<0.0001*
	Unmarried	59 (33.15)	
	Widow/Widower	3 (1.67)	
Education	Primary	83 (46.63)	<0.0001*
	Secondary	47 (26.4)	
	Higher secondary	35 (19.66)	
	Graduation	11 (6.18)	
	Above graduation	2 (1.12)	

Pre-intervention score in the survey questionnaire was 18.93 ± 5.10 which significantly increased after the intervention to 25.06 ± 4.35, as shown in Table 2.

**Table 2:** Score of the survey before and after intervention (n = 178). *: Statistically significant p-value of paired t-test. SD: standard deviation; SEM: standard error of the mean

Score statistics	Pre-intervention	Post-intervention	P-value
Mean	18.93	25.06	<0.0001*
SD	5.10	4.35	
SEM	0.38	0.33	

When we enquired about the vaccination status before and after the intervention, we found that the number of individuals receiving vaccination significantly increased after conducting the educational program, as shown in Table 3.

**Table 3:** Vaccination status before and after the educational program. *: Significant p-value of one-sample proportion test; †: Took any one of the vaccines provided by the Indian government; ‡: Total number of individuals who received the second dose; not included in the statistical test.

Vaccination status†	Pre-intervention	Post-intervention	P-value
No vaccine	95	17	<0.0001*
First dose	83	161	
Second dose	0	112‡	

## Discussion

With an aim to observe the effect of an educational program on improving the knowledge about COVID-19 vaccination, we found that the educational program had a positive impact on improving the knowledge about COVID-19 vaccination in the study population. The importance of this study lies in the potential implications for future interventions and programs.

Vaccine hesitancy due to improper knowledge is a significant public health issue. People who are hesitant to vaccinate may also have concerns about the potential side effects of vaccines or may hold misconceptions about the diseases that vaccines are designed to prevent [6,7]. Vaccine hesitancy due to improper knowledge can have serious consequences, including outbreaks of vaccine-preventable diseases, increased healthcare costs, and the unnecessary suffering and death of individuals who could have been protected by vaccination. To address vaccine hesitancy due to improper knowledge, it is important to provide accurate information about vaccines and address any concerns or misconceptions that individuals may have [8]. Our study suggests that an educational program would help increase the knowledge about COVID-19 vaccination.

Spreading awareness about the benefits of vaccines could also build confidence through changes in the education curriculum. Providing local language communication of vaccine benefits backed by scientific evidence of vaccine safety and efficacy is perhaps one of the most important ways to increase people’s trust and confidence in vaccinations [9]. A door-to-door campaign in vaccine-hesitant areas might be crucial not only to dispel myths and promote vaccines but also to address people’s concerns and restore confidence in vaccines and their government before more hesitancy spreads to other regions [10]. However, when door-to-door visits are not feasible, a small group of the population can be chosen to disperse knowledge as we did in our study.

Several studies have investigated the factors contributing to vaccine hesitancy in India, including misinformation, lack of trust in the government and healthcare providers, and concerns about the safety and efficacy of the vaccine [11-14]. The rural population has faced significant challenges in accessing pandemic and vaccine information during the COVID-19 pandemic. Several factors have contributed to this, including limited access to technology and the internet, language barriers, and a lack of public health messaging targeted specifically at rural populations. One of the primary challenges facing rural populations is limited access to technology and the internet. Many rural areas lack high-speed internet connectivity, making it difficult for individuals to access online resources and information about the pandemic and vaccines. Additionally, many rural residents may not have access to smartphones or other devices that would allow them to access information digitally [15]. Language barriers also pose a significant challenge to rural populations in accessing pandemic and vaccine information. In some areas, residents may speak languages other than the official language of the country, which can make it difficult for them to access information that is only available in the official language. This can lead to misunderstandings or confusion about the pandemic and vaccines. A lack of public health messaging targeted specifically at rural populations is another challenge.

To address these challenges, primary care physicians and healthcare workers may play a vital role. They can directly participate in health educational programs to disseminate information on the importance of vaccination to prevent diseases or reduce the severity of diseases. Furthermore, video-based educational programs in the local language or dialect may be an effective tool for disseminating public health-related issues.

Limitations

This study has several limitations. Although we initially recruited 318 participants, we could conduct completed pre- and post-intervention surveys in 178 individuals, and the result reflected in this study is of the final sample only. Furthermore, this study did not have a control. Hence, we are unable to comment on the improvement of vaccination knowledge and actual receiving of vaccination without intervention. In addition, this study was conducted in a particular block in a district of Jharkhand and may lack extension to other populations.

## Conclusions

A structured educational program with videos in the local language may help increase knowledge about vaccination that we found for COVID-19. The educational program can influence people for getting vaccinated in a timely manner. In rural areas where vaccine hesitancy is high, similar educational programs can be conducted to increase the rate of vaccination. The finding of this study can be taken as a reference for any future vaccination program.

## Appendices

Appendix 1

The surveyor-administered questionnaire used in this study.

**Table 4:** Questionnaire used for the survey.

Question	Score
Do you know what COVID-19 is?	Wrong = 0, Correct = 1
Does COVID-19 affect the human body?	Wrong = 0, Correct = 1
What are the symptoms of COVID-19 disease?	No correct symptom = 0, one correct symptom = 1, two correct symptoms = 2, three correct symptoms = 3, four correct symptoms = 4
Is COVID-19 a life-threatening disease?	Wrong = 0, Correct = 1
How to prevent COVID-19 disease?	No correct preventive method = 0, one correct preventive method = 1, two correct preventive methods = 2, three correct preventive methods = 3, four correct preventive methods = 4
Is vaccination against COVID-19 a type of preventive measure to protect oneself from the ill effects of the infection?	Wrong = 0, Correct = 1
Does the vaccine increase your body’s capacity to fight against the infection?	Wrong = 0, Correct = 1
Has India started the COVID-19 vaccination program?	Wrong = 0, Correct = 1
Is the COVID-19 vaccine free?	Wrong = 0, Correct = 1
How many doses of COVID-19 vaccine are required?	Wrong = 0, two doses = 1, two doses + booster dose = 2
How much of a time gap should be between doses of vaccine?	Wrong = 0, Correct = 1
How many types of vaccines are available?	Wrong = 0, Correct = 1
Do you get optimum protection against COVID-19 infection with one dose of vaccine?	Wrong = 0, Correct = 1
Do you get optimum protection against COVID-19 infection with only two doses of vaccine?	Wrong = 0, Correct = 1
A booster dose of the COVID-19 vaccine gives better protection.	Wrong = 0, Correct = 1
Can we still get infected with COVID-19 after receiving two doses of the vaccine?	Wrong = 0, Correct = 1
Post-vaccination, one may have side effects	Wrong = 0, Correct = 1
Can you name some side effects (SE)?	one correct SE = 1, two correct SEs = 2, three correct SEs = 3, four correct SEs = 4
Where do you have to report if you face any side effects of the COVID-19 vaccine?	Wrong = 0, Correct = 1
Can you take a vaccine even after having comorbidities like diabetes, hypertension, and thyroid-related issues?	Wrong = 0, Correct = 1
Should you take the vaccine even after getting infected with COVID-19?	Wrong = 0, Correct = 1
Is COVID-19 vaccination required for protecting oneself from the adverse effects of COVID-19 infection?	Wrong = 0, Correct = 1
Maximum score = 32	

Appendix 2

Content of the educational video (part of the intervention)

·       Definition and introduction of COVID-19 and the infective agent

·       Effects of COVID-19 on the human body

·       Preventive methods - COVID-19-appropriate behavior

·       Introduction to vaccines

·       Importance of vaccines

·       What immunity is and how the immune system works

·       Method of action of vaccines

·       COVID-19 vaccination program in India

·       Dose schedule

·       Eligibility and contraindications

·       Possible side effects of vaccines

·       Risks of not getting vaccinated

·       Possible side effects of the COVID-19 vaccine

·       What an individual should do if they experience adverse reactions to the COVID-19 vaccine
